# Sequence and organization of the complete mitochondrial genome of the blackfly *Simulium variegatum* (Diptera: Simuliidae)

**DOI:** 10.1080/23802359.2016.1209091

**Published:** 2016-11-22

**Authors:** John C. Day, Hyun S. Gweon, Rory J. Post

**Affiliations:** a Centre for Ecology and Hydrology (CEH) Wallingford, Wallingford, Oxfordshire, UK;; b School of Natural Sciences and Psychology, Liverpool John Moores University, Liverpool, UK;; c Disease Control Department, London School of Hygiene & Tropical Medicine, London, UK

**Keywords:** Blackfly, Diptera, mitochondrial genome, Simuliidae, *Simulium variegatum*

## Abstract

The complete mitochondrial genome of the European blackfly, *Simulium variegatum* Meigen, 1818 was sequenced using a combined Illumina and Sanger sequencing approach. Using the known sequence of *Chironomus tepperi* Skuse, 1889 (Chironomidae) homologous NGS reads were identified and assembled. The genome is 15,367 bp in length and includes 13 protein-coding genes, 2 ribosomal RNA genes, 22 transfer RNA genes and a control region. Gene order resembles that of the ancestral dipteran gene arrangement. The base composition of the genome is A (37.6%), T (35.3%), C (15.8%) and G (11.3%). The control region between 12S rRNA and tRNAIle is composed of 362 bp with no obvious repetitive motifs.

Blackflies belong to the family Simuliidae, a group of highly specialized aquatic insects. Adult females search for a blood meal and are notorious vectors of blood borne disease, most notably river blindness, onchocerciasis. *Simulium variegatum* (Meigen, 1818) is commonly found in small streams to large rivers throughout Ireland and the north and west of Britain. *S. variegatum* takes blood from dogs and livestock as well as man (Davies & Williams 1962). Their range extends from Algeria and Portugal in the West, throughout Europe through to Iran and the Caucasus in the East and with fourteen synonyms may be composed of a species complex (Adler & Crosskey 2015).

The *S. variegatum* specimen used in this study was collected from the River Alyn at Llandegla, Denbighshire (53.063214, -3.201966) on the 25 June 2014 as a pupa and preserved in absolute ethanol. DNA was sequenced on a HiSeq 2500 sequencer (Illumina) (San Diego, CA) as part of an environmental genomics project. The pupal cocoon was archived at CEH under the voucher code CEHSIMJD0822. The genome was assembled with Geneious 8.1.7 (Auckland, New Zealand) (Kearse et al. 2012) using the mitogenome of *Chironomus tepperi* (Acc. No. JN861749) as a reference. The first draft genome was used as a search query to determine additional reads in the 10 million read database. Finally, PCR and Sanger sequencing was used to validate the accuracy of ambiguous regions located in the non-coding region. A combination of primers (available from the author) was developed to amplify the entire control region of the *S. variegatum* mitochondrial genome. The mitogenome was annotated for CDS, 16S, 18S and the control region by comparison with the *C. tepperi* genome and using the ORF finder function in Geneious and the MITOS web server (Bernt et al. 2013). For tRNA prediction, the programs ARWEN v1.2.3 and tRNAscan-SE 1.21 were used (Lowe & Eddy 1997; Laslett & Canback 2008). The complete mitogenome of *S. variegatum* was assembled using a combination of Illumina Hiseq (San Diego, CA) and Sanger sequences. The final assembly contains 9593 Illumina sequences, 0.2% of the total dataset with an average sequence coverage of 113. The mitochondrial genome includes 13 protein-coding genes, 22 transfer RNA genes, 2 ribosomal RNA genes and a control region between 12S rRNA and tRNAIle, composed of 361 bp containing no apparent repetitive units. The base composition of the genome was determined to be A (37.6%), T (35.3%), C (15.8%) and G (11.3%) with a GC content of 27.0%. The annotated mitogenome, with a length of 15,367 bp, is available online at NCBI (GenBank accession number KU252587) and shares the highest sequence identity to the mitochondrial genome of *Simulium aureohirtum* (Figure 1).

**Figure 1.**
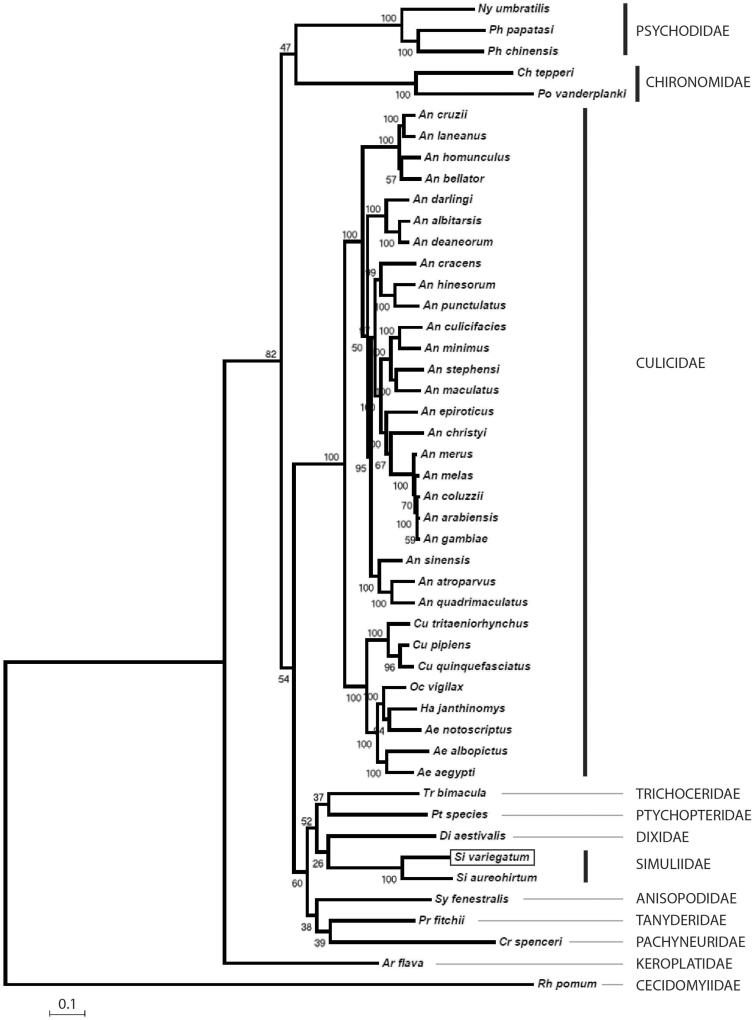
A maximum likelihood phylogenetic tree of the mitochondrial genomes *Simulium variegatum* (shown boxed) and forty six further Nematocera taxon sequences retrieved from GenBank. Sequence alignments were conducted using ClustalW and the tree constructed using PhyML with 100 bootstrap replicates using the GTR + I + G model of substitution. An alignment of 14114 characters was used for the analysis incorporating the following sequences: *Aedes aegypti* EU352212; *Aedes albopictus* AY072044; *Aedes notoscriptus* KM676218; *Anopheles albitarsis* HQ335344; *Anopheles arabiensis* KT382816; *Anopheles atroparvus* KT382817; *Anopheles bellator* KU551287; *Anopheles christyi* KT382818; *Anopheles coluzzii* KT382819; *Anopheles cracens* JX219733; *Anopheles cruzii* KJ701506; *Anopheles culicifacies* KT382820; *Anopheles darlingi* GQ918272; *Anopheles deaneorum* HQ335347; *Anopheles epiroticus* KT382821; *Anopheles gambiae str. PEST G3* L20934; *Anopheles hinesorum* JX219734; *Anopheles homunculus* KU551283; *Anopheles laneanus* KU551288; *Anopheles maculatus* KT382822; *Anopheles melas* KT382823; *Anopheles merus* KT382824; *Anopheles minimus* KT382825; *Anopheles punctulatus* KT382826; *Anopheles quadrimaculatus* L04272; *Anopheles sinensis* KT218684; *Anopheles stephensi* KT382827; *Arachnocampa flava* JN861748; *Chironomus tepperi* JN861749; *Cramptonomyia spenceri* JN861747; *Culex pipiens pipiens* HQ724614; *Culex quinquefasciatus* GU188856; *Culex tritaeniorhynchus* KT852976; *Dixella aestivalis* KT878382; *Haemagogus janthinomys* KT372555; *Nyssomyia umbratilis* KP702938; *Ochlerotatus vigilax* KP721463; *Phlebotomus chinensis* KR349297; *Phlebotomus papatasi* KR349298; *Polypedilum vanderplanki* KT251040; *Protoplasa fitchii* JN861746; *Ptychoptera species* JN861744; *Rhopalomyia pomum* GQ387649; *Simulium aureohirtum* KP793690; *Sylvicola fenestralis* JN861752; *Trichocera bimacula* JN861750.

We provide the first complete mitochondrial genome from a European simuliid. Little is known about the molecular biology of blackflies in comparison with other medically important Diptera. A recent study illustrated the paucity of blackfly sequences available in GenBank in comparison to other Diptera (Adler et al. 2010). However, a blackfly BAC library is now available (Crainey et al. 2010) and a current blackfly genome project is in development (Brockhouse 2015). The availability *S. variegatum* mitogenome will provide a useful resource for understanding Simuliidae evolution.
